# Cancer Prevention and Screening for People Experiencing Homelessness: Co-Designing the Health Navigator Model

**DOI:** 10.5334/ijic.9293

**Published:** 2025-11-11

**Authors:** Alejandro Gil-Salmerón, Christina Carmichael, Tobias Fragner, Maria Moudatsou, Ioanna Tabaki, Jaime Barrio Cortes, Ascensión Doñate-Martínez, Lee Smith, Igor Grabovac

**Affiliations:** 1 International Foundation for Integrated Care, Oxford, UK; 2 Complutense University of Madrid, Madrid, Spain; 3 School of Psychology, University of East Anglia, Norwich, UK; 4 Centre for Health, Performance and Wellbeing, Anglia Ruskin University, Cambridge, UK; 5 Department of Social and Preventive Medicine, Centre for Public Health, Medical University of Vienna, Vienna, Austria; 6 PRAKSIS-Programs of Development, Social Support and Medical Cooperation-Non-Profitable Association, Athens, Greece; 7 Foundation for Research and Biosanitary Innovation in Primary Care, Madrid, Spain; 8 Camilo José Cela University, Madrid, Spain; 9 Biomedical Data Science Lab – ITACA Institute, Universitat Politecnica de Valencia, Valencia, Spain

**Keywords:** homelessness, cancer prevention, cancer screening, patient navigation, patient empowerment, co-design

## Abstract

**Background:**

People experiencing homelessness (PEH) face major barriers to accessing healthcare, including cancer preventive services, which results in increased cancer morbidity and mortality. However, tailored integrated care interventions addressing these disparities are scarce.

**Methods:**

Using a qualitative, participatory approach, seven focus group discussions were conducted with 15 PEH and 41 health and social care professionals in Austria, Greece, Spain, and the UK. Data were thematically analysed using a framework based on ten core components of navigation interventions.

**Results:**

Collaborative discussions led to a consensus on the Health Navigator Model (HNM), designed to improve cancer prevention for PEH. This model introduces “health navigators” from health and social care backgrounds to identify health needs, raise cancer awareness, coordinate healthcare access, and provide practical support. Thematic analysis ensured consistency across countries, shaping a person-centred approach. Comprehensive training and supervision were identified as critical for the effectiveness of the HNM.

**Conclusion:**

The co-design approach allowed PEH and professionals to actively shape the intervention, addressing gaps in cancer prevention. The HNM offers a structured, internationally consistent model that could bridge access gaps in cancer care for PEH. Further research using implementation science frameworks is needed to evaluate its effectiveness in real-world settings.

## Introduction

The European Union has witnessed a 30% increase in homelessness since 2018, affecting over 1,286,691 individuals in Europe and signalling a severe social crisis [1]. Compared with the housed population, people experiencing homelessness (PEH) face significantly higher morbidity and mortality rates, with cancer being the second leading cause of death [2]. Contributing factors to this disparity include limited access to healthcare and the prevalence of cancer risk factors within this community, such as tobacco and alcohol use [3,4,5]. Despite their heightened risk, PEH encounter obstacles in accessing cancer prevention initiatives [6,7], whereas a recent qualitative study with PEH and healthcare professionals revealed a need for tailored cancer prevention initiatives, emphasising accessible health education and building trusting relationships between professionals and PEH [8].

According to Lennox-Chhugani [9], adopting a population health management approach through integrated care enables health and care systems to shape and address the social determinants of population health. Navigational approaches have been shown to reduce inequalities and mitigate screening barriers at systemic, provider, and individual levels [10,11]. Patient navigation is a promising model for reducing health disparities and ensuring timely access to quality cancer care [12]. It involves dedicated navigators guiding patients through the healthcare system. Evidence indicates that patient navigation can offer potential benefits for PEH, with improved access and health outcomes, prompting a recommendation for global exploration beyond North America to assess feasibility, efficacy, and scalability, advocating for the adoption of patient navigation while emphasising the need for further international research [13]. While patient navigation provides crucial support, it is also essential to recognise the role of patient empowerment in enhancing users’ healthcare experiences. Patient empowerment is defined as a process and an outcome through which individuals gain self-confidence and self-efficacy to actively participate in their own healthcare; ultimately, exercising power over decision-making concerning their treatment is crucial for overcoming the powerlessness and dependency often associated with the living situations of PEH [14]. In this context, patient empowerment interventions have also improved health outcomes, including greater care satisfaction and treatment adherence, simultaneously reducing health disparities [15].

Conceptually, integrated care encourages health and care providers to partner with individuals to co-design and deliver person- and community-centred care that provides people with the high-quality care and support they need at the right place and time [9]. Previous studies have demonstrated that participatory methods lead to healthcare services that are more closely aligned with the actual needs of both patients and professional care teams, fostering innovative and effective care solutions [16,17,18]. This paper reports on a study aimed at co-designing the novel Health Navigator Model (HNM), which embeds and intertwines patient navigation and patient empowerment principles to facilitate cancer prevention for PEH.

## Method

### Study Design

This research was conducted within the Horizon 2020-funded project “Cancer Prevention and Early Detection among the Homeless Population in Europe: Co-adapting and Implementing the Health Navigator Model” (CANCERLESS). The CANCERLESS project aimed to prevent cancer and facilitate early cancer diagnoses for PEH by delivering integrated person-centred care co-designed to address health inequities and facilitate timely access to quality cancer prevention and screening services. After performing a systematic scoping review [13] and two exploratory qualitative studies [8,19] to understand navigational approaches and contextual barriers in cancer care for PEH, a participatory study using focus groups was conducted with PEH as well as health and social care providers and service managers working in the homelessness sector.

### Procedure

Participants were recruited through partnering health and social care organisations that provide services to PEH. This included professionals working directly with PEH, as well as individuals currently engaged with these services, such as shelter residents and regular users of health or support programs. The focus groups were held both in person and online (due to COVID-19 restrictions) between December 2021 and January 2022, and were conducted by trained research team members. After initial input from the trained facilitators outlining the background and aim of the HNM, the focus groups lasted between two and three hours. To ensure consistency across countries, a structured framework based on the ten core components of navigation interventions, initially outlined by DeGroff et al. [20], guided the focus group discussions. These components cover various aspects of the intervention: program goals, community characteristics, point of intervention, setting(s) of intervention, range of services offered by health navigators (HNs), HN background and qualifications, channels of communication, HN training and supervision as well as evaluation measures. Participatory activities such as mind-mapping and ‘diamond nine’ ranking exercises [21] were employed to facilitate interaction, organise ideas, and foster innovation.

### Data Analysis

Focus group discussions were audio or video recorded and transcribed verbatim in their respective languages, either manually or through appropriate software, and internally checked for quality. The transcripts were analysed via both inductive and deductive strategies. Initially, researchers employed an inductive thematic approach [22] to code the transcripts, systematically capturing relevant information. These codes were then synthesised and organised deductively into a predetermined thematic framework [20]. Researchers involved in the analysis met at regular intervals and discussed coding strategies to ensure the validity of the process.

Based on this analytical procedure, researchers in each country summarised their focus group discussions, highlighting key decisions, outcomes, and themes within the given framework. These summaries were then integrated through cross-comparison and collaborative discussion to form an overall co-adapted framework for the HNM.

### Ethical Aspects

The study received approval from the Ethics Committee of the Medical University of Vienna (coordinating institution), with additional approval obtained from local partner institutions. All participants provided written informed consent before participating in any focus group activities. In the case of online focus groups, the informed consent forms were sent prior to the focus group, with participants gaining access to the online platforms once they returned the signed forms. Ground rules were set for respectful conversations, and the data were stored securely in compliance with the General Data Protection Regulation, with anonymised transcripts and encrypted files.

## Results

Seven focus group discussions were conducted across four European countries (Austria, Greece, Spain, and the UK), resulting in a total sample size of 56 participants. These included 41 professional stakeholders and 15 PEH (Table 1).

**Table 1:** Details of co-design focus group discussions across Austria, Greece, Spain (Madrid), and the UK, including the meeting modalities used and the composition of participants in each session.


LOCATION	MODALITY	PARTICIPANTS

**Austria**	Online videoconference	3 social/support workers, 2 peer support workers, 2 general practitioners, 1 homelessness social service manager, 1 healthcare manager, 1 public health researcher

**Greece**	Online videoconference	14 stakeholders (NGOs, health and social care professionals, public health experts, local government representatives), 2 individuals with experiences of homelessness

**Spain (Madrid)**	In-person, Videoconference	In-person: 9 PEH. Online: 9 professionals (doctors, nurses, oncologists, social workers)

**UK**	Online videoconference, In-person	In-person: 4 male service users, 1 support worker. Online: 7 service managers and representatives of a charitable organization.


The results are presented according to the ten core components of health navigation, following the DeGroff et al. framework [20]. While no formal hypotheses were applied, expectations were informed by prior literature, contextual knowledge of national systems, and insights from previous phases of the project. This structure allows for comparative interpretation of how different healthcare and social care systems prioritised and adapted each component [13].

### 1. Program Goals

Participants across the four countries identified various goals for the HNM regarding cancer prevention interventions among PEH, including overcoming healthcare and welfare system barriers, raising awareness of cancer prevention, centralising services (Austria), enhancing cooperation between PEH and healthcare services, increasing cancer screening rates, addressing fundamental needs such as accommodation (Greece), reducing healthcare barriers, increasing cancer detection rates, involving patients in their care process (Spain), improving understanding of cancer prevention, enhancing relationships between users and healthcare services, increasing cancer screening rates, and delivering person-centred care (UK), collectively highlighting the importance of comprehensive interventions to address healthcare barriers, raising cancer awareness, and improving access to services for PEH:

*“I think that’s got to be your number one thing, to get it [information] out there, so that people know and are aware of it [cancer prevention], so that they can do something about it if they choose.”* (PEH, UK)

The diversity in focus across countries reflects structural and policy-level variations in how homelessness is addressed. UK participants’ concern with transient populations and Austrian participants’ emphasis on high-needs subgroups illustrate differing service models and outreach strategies.

### 2. Community Characteristics

Various perspectives have emerged across countries regarding community characteristics relevant to designing cancer prevention interventions for PEH. In Austria, participants emphasised the importance of targeting subgroups such as those with challenging behaviours or complex needs, smokers, and men, advocating for low-threshold services. Participants in Greece highlighted designing interventions for people experiencing any form of homelessness and engagement strategies for individuals diagnosed with cancer, while those in Spain stressed the need for intervention accessibility and prioritising assistance for disadvantaged groups, including active outreach to ‘hidden’ populations. Similarly, participants in the UK underscored offering interventions to PEH, focusing on engaging men and transient PEH owing to specific health needs and less awareness. Common themes were identified, emphasising the importance of targeting subgroups within PEH, ensuring intervention accessibility, and prioritising assistance for at-risk populations, all aimed at designing effective cancer prevention interventions:

*“That hidden sort of homelessness… transient populations [who] may be moving for various reasons from different locations and so are missing, you know, even if they’ve registered with a GP, they might miss opportunities.”* (Health provider representative, UK)

National system structures likely influence where interventions are seen as most effective. Austria’s preference for early integration aligns with more formal entry points into care, while the UK’s and Greece’s focus on post-diagnosis support underscores the importance of flexible, relationship-driven approaches.

### 3. Point of Interventions

In Austria and UK there was a general agreement on the critical points in the care continuum where the HNM can make a meaningful impact. They emphasised the importance of preventive health navigation to avert severe health consequences as well as immediate integration of PEH into the healthcare system post-diagnosis:

*“If I can integrate a person into a social network before he or she becomes ill, (…) it is more likely that this person will make use of or seek out certain services later on.”* (Peer support worker, UK)

The participants in Greece highlighted the significance of trust between HNs and PEH, as well as the role of peers in encouraging engagement with cancer prevention:

*“When you have a former [person experiencing homelessness] and a current [person experiencing homelessness], it carries a lot of importance; it gives him hope, it gives him confidence that I will get through this.”* (Professional representative, Greece)

In Spain, emphasis was placed on the necessity for HNs to be actively involved in outreach activities for PEH and sustain their participation in the intervention. Similarly, participants in the UK highlighted the importance of HNs being embedded within familiar spaces of PEH and the comprehensive involvement of HNs across the entire cancer care continuum. Across countries, the importance of preventive health navigation, trust-building between HN and PEH, and comprehensive HN involvement across the continuum of care has emerged as a constant theme in the analysis.

### 4. Settings of Intervention

The findings regarding the settings of interventions for cancer prevention among PEH reveal differing approaches and considerations across countries. Participants in Austria emphasised establishing outreach services and embedding HNs in health and social care facilities, whereas in Greece the focus was placed on outreach activities and mobile healthcare units for initial engagement, with referrals to formal clinical settings as necessary. Participants in Spain prioritised using familiar spaces and offered telephone communication options for active involvement, as professionals in the UK highlighted the central role of peers in engaging PEH with cancer prevention activities keeping in mind the involvement of more formalised clinical settings in delivering preventative care and noting the earnestness of the approach:

*“Shifting to a proper medical facility as soon as possible just so it underlines the gravity of the situation, it makes it that much more real.”* (Homelessness service manager, UK)

The comparison highlighted the importance of HNs being adaptable and accessible in locations frequented by PEH, such as shelters/hostels or day centres, and in services that effectively reach these groups, such as mobile healthcare units or outreach teams.

### 5. Range of Services to be Offered by HNs

The cross-country comparison regarding the services to be offered by HNs indicated differing perspectives and priorities. Participants in Austria emphasised a need for a clearly defined scope of activities for HNs, focusing on facilitating access to services and providing practical assistance while avoiding tasks outside the realm of cancer screening and prevention:

*“I just wonder how much this person is supposed to do. (…) It would be more important to work in an interdisciplinary way and to know who can do which work. Because there are many professionals in the field who can do this, and for me it is somehow about a clear definition of what the services of this navigator are and what they do not have to do.”* (NGO representative, Austria)

In Greece, a personalised approach tailored to individual needs was underscored, including practical assistance and additional services for cancer diagnosis. At the same time, those in Spain identified common themes such as sharing information on cancer, addressing basic needs, and supporting healthcare management:

*“What these people need most is to be able to have transportation, to help us with appointments; practical, real help, something that can be used and useful to us.”* (PEH, Spain)

In the UK, differing views on the services offered were observed, emphasising flexibility, responsiveness, and role adaptability over time. PEH across all countries stressed the importance of practical assistance, particularly concerning transportation and appointment attendance:

*“Making sure we have a phone so they can contact them [users], make sure they’ve got the right date for the appointment … they might not have transport, they might not have a bus pass, then actually attending the appointment, that’s where the navigator should come in, then when they have to go back for another check-up if they need one, that sort of thing.”* (PEH, UK)

In summary, the standard services should focus on facilitating access to services, providing practical assistance tailored to individual needs (assisting with transportation and accompanying PEH to appointments), addressing basic needs, and offering flexibility and adaptability over time.

### 6. HN Background and Qualifications

The qualifications and characteristics deemed essential for HNs varied slightly across the four countries. In Austria, participants emphasised personal qualities such as empathy, social competence, and the ability to set personal boundaries alongside prerequisites such as basic education in the psychosocial or medical field. The participants in Greece emphasised the involvement of a multidisciplinary team, including primary care physicians, nurses, and social/support workers, along with the role of individuals with lived experience of homelessness as mediators:

*“I think an important factor that helps to reach out to the homeless population is peer support workers … people who have had experience with homelessness. These people should be the mediators and support, help with the communication of homeless with medical staff. A mobile unit is not easy for the homeless in terms of visiting a doctor. There should be a mediator who could more easily reach these people.”* (Professional representative, Greece)

In Spain, participants leaned towards support or social workers as HNs, highlighting the importance of understanding local resources and healthcare delivery while acknowledging the value of a multidisciplinary team:

*“I see as necessary that there is a health figure with a focus on community and public health and also a social worker as part of this team who knows about the benefits and resources.”* (Mental health nurse, Spain)

In the UK, there was a preference for HNs with direct experience of homelessness or those already working with PEH. Interpersonal skills such as empathy were emphasised over cancer-specific expertise. However, there was also a recognition of the need for health professionals’ involvement and practical assistance in supporting HNs:

*“If they [current homelessness support workers] were HNs, they would have tremendous strength because they’re already embedded.”* (PEH, UK)

The commonalities across countries underscore critical aspects of the HN background. The role of HN can be taken by individuals with lived experience of homelessness or professionals with working experience in this field. The preferred professional background should be within the psychosocial or medical sectors. The discussions also highlighted the HNs’ need to work and collaborate in a care network. Diverging views on required qualifications suggest differences in professional hierarchies and workforce capacity across countries. Austria’s clinical emphasis differs from the UK’s emphasis of peer support and lay navigators involvement.

### 7. Channels of Communication Between Users and HN(S)

The preferred communication channels between PEH and HNs varied, reflecting the diverse needs and contexts of PEH in each country, with the exception of the importance of regular in-person meetings which was acknowledged in all sessions. In Austria, participants emphasised the importance of providing multiple access points to the navigation service, suggesting the establishment of a central coordination office to facilitate easy access for PEH. They also highlighted the role of HNs as intermediaries between organisations to address individual needs comprehensively:

*“And I would also like it if there were a variety of possibilities for the [health navigation] service. That is, if it were possible to get to the service not only through one channel … If we could outsource this issue [subsequent care and support for PEH] to someone else, it would, of course, be great because we probably cannot solve it in social work and that is a problem.”* (Homelessness service manager, Austria)

In Greece, participants recognised the challenge of building trust with PEH and advocated for consistent face-to-face contact between HNs and users, ideally every week. Similarly, in Spain, participants preferred face-to-face meetings, acknowledging the need for periodic interactions to deepen their understanding of user needs. However, they also acknowledged the potential benefit of mobile phone communication between meetings. In the UK, participants stressed face-to-face meetings as the primary mode of communication, supplemented by occasional phone check-ins, reflecting the recognition that digital literacy and access can be low among some PEH. Both informal drop-in sessions and formal appointments were proposed, with the frequency and style of communication being user-led.

*“Like a weekly drop-in but a more casual one, so if the navigators were at the day centre one day a week if that person sat there and they could be working away on a laptop or having a cup of tea helping out just doing alternative voluntary stuff, then the people come to them.”* (Homelessness service manager, UK)

### 8. HN Training

The participants in Austria emphasised the importance of comprehensive pre-practice training for HNs and proposed compulsory training covering basic medical and communication skills, including topics such as local health and social care systems, cancer education, and motivational interviewing. In Greece, participants described training for HNs as crucial, with suggested areas including homelessness, communication skills, cancer education, and understanding the local context and resources, highlighting the need to educate staff in homelessness services and public hospitals. Similarly, in Spain, participants stressed comprehensive training covering cancer education, the local context, and mental health, with participants experiencing homelessness underscoring the importance of effective communication and engagement training:

*“The navigator should be someone with experience in the field of social exclusion and someone from the health world… they have to be people who can understand the health process and the level or stage the person is at. They should be professionals with psychosocial training and public health training.”* (Mental health nurse, Spain)

Training was consistently viewed as essential across countries, though its focus and structure varied depending on the HN background and the national context. In the UK, training priorities included mental health, substance use, cancer education, and motivational interviewing, with an emphasis on experiential learning and access to ongoing resources. These expectations reflect the community-oriented structure of the UK’s health and social care system. In contrast, Austria favoured more formalised and structured training pathways, aligning with its professionalised and institutionalised care system. Overall, training expectations appear to mirror national education frameworks and the complexity of care navigation required.

In conclusion, training for HNs should include foundational medical and communication skills, cancer awareness, understanding of local health and welfare systems, and effective engagement strategies with PEH. Ongoing training and structured support were widely recommended to ensure HNs are equipped to adapt to evolving needs in practice.

### 9. HN Supervision

Participants in Austria emphasised the importance of supervision for HNs, suggesting both case-specific supervision sessions and peer coaching for exchanging experiences. In contrast, in Greece and Spain, there was consensus among participants on the necessity of coaching and ongoing supervision by professionals with relevant expertise in working with PEH, with a focus on administrative, social, and health aspects.

In the UK, supervision was seen as crucial, with a proposed model including formal meetings, informal catchups, and immediate debriefs.

*“I think that access for them [HNs] to debrief when there’s, you know, something that’s very emotive is really important.”* (Health service representative, UK)

Although supervision was recognised as a critical component across all participating countries, the preferred approaches varied. These differences may reflect broader institutional norms and organisational cultures. Countries with more hierarchical systems, such as Austria, emphasised structured monitoring and formal oversight. In contrast, others favoured more collaborative and reflective supervision models, including peer coaching and case-specific sessions.

### 10. Evaluation Measures

In Austria, participants discussed evaluation measures, suggesting pre/post and international comparisons. The quantitative data proposed to be collected included case numbers served, and contact frequencies, whereas the qualitative data to be included were quality-of-life surveys and qualitative interviews. In Greece, the focus was on continuously monitoring user needs and assistance, along with feedback from all involved parties. Spain highlighted tracking health outcomes and cancer screening timeliness, focusing on user feedback and service provision details:

*“Timeliness of cancer screening is paramount. It is important that they [i.e., appointments] are requested or that they make an appointment with you [i.e., the HN] as soon as possible in order to carry them out and that people learn about their own health status.”* (PEH, Spain)

In the UK, measuring cancer screening outcomes, cancer education, user engagement, and HN-user relationships were prioritised, and the impacts on primary care engagement and local stakeholders’ feedback by the participants were assessed:

*“It’s important for you to measure, after that initial engagement, how many people actually follow through.”* (PEH, UK)

*“Their [PEH] level of understanding … You know, what they gained from an education and information standpoint from the navigators and this project.”* (Health service representative, UK)

Evaluation measures discussed across the countries included pre- and post-intervention comparisons, cross-country analyses, ongoing monitoring of user needs, tracking of health outcomes, and assessments of impact on primary care engagement, supplemented by local stakeholder feedback. The preferences for evaluation approaches appear to reflect national accountability structures. For instance, participants in Austria emphasised the use of measurable indicators, aligning with its formal and standardised health system evaluation frameworks. In contrast, participants in other countries, particularly the UK and Greece, showed a greater interest in outcome narratives and qualitative feedback, reflecting more community-based approaches to assessing intervention effectiveness.

## The Health Navigator Model

The HNM for cancer prevention interventions among PEH aims to overcome users’ healthcare barriers, increase cancer awareness, and improve access to PEH healthcare services. HN services should encompass a range of comprehensive support, addressing practical assistance, including providing patient education, appointment scheduling, transportation assistance, financial counselling, and emotional support. The point of intervention in the cancer care continuum is from awareness to early detection and primary care engagement, ensuring PEH can effectively access cancer prevention services. The community characteristics of PEH, such as their unique needs, demographics, and cultural and socioeconomic factors, are inherently considered in the intervention. The intervention setting includes places where face-to-face communication is feasible, such as shelters, community centres, or clinics.

The HN role can be taken on by health and social care professionals such as social workers, as well as by individuals with lived experience of homelessness or professionals working with PEH receiving additional relevant training to serve the target population effectively. Effective face-to-face communication between HNs and PEH is crucial, and in-person meetings should ensure effective information exchange and support. It is essential to establish ongoing supervision and support for HNs, ensuring they receive necessary oversight and emotional support, which helps maintain the quality and consistency of care provided. Continuous monitoring of PEH needs and assistance, tracking health outcomes, and evaluating the impact on primary care engagement and local stakeholders’ feedback is essential. This involves ongoing formative and summative evaluation measures to assess the effectiveness of the intervention.

## Discussion

This is the first international comparative perspective to co-design the Health Navigator Model (HNM), a person-centred, integrated care model for cancer prevention for PEH across four health and social care systems in Europe. By actively involving PEH, healthcare professionals, and service managers in the design process, we co-designed a model that is adaptable, flexible, and responsive to the unique needs of this population. The study’s results are essential for future implementation, as they have directly informed the project’s transition from design to the practical, real-world application of the HNM.

The international differences observed in the co-design process reflect deeper structural features of national health and welfare systems. Austria’s emphasis on formal coordination and role clarity is characteristic of Bismarckian models, which prioritise structured, insurance-based care with strong institutional oversight. In contrast, the UK and Greece, both influenced by Beveridgean principles, favoured more relationship-based and flexible approaches, often rooted in community-level engagement and shaped by experiences of system fragmentation. Spain with similarly aligned ideals in its focus on accessibility and outreach, though its highly decentralised healthcare governance introduces significant regional variability in service provision. These typological distinctions might help explain the varied priorities and implementation preferences across countries, underscoring the need for context-sensitive adaptations of the HNM to ensure its relevance and effectiveness within differing national systems.

This work builds upon a literature review on navigational approaches for disadvantaged groups, particularly PEH [13], and qualitative research exploring healthcare barriers and implications for cancer prevention interventions for PEH [8]. Our study successfully co-designed a care model addressing the barriers to cancer prevention for PEH, as identified by Jeleff et al. [5], leading to the development of the novel HNM, which is adaptable to various health and social care contexts. The co-design process incorporated the voices of PEH, allowing them to express their needs and preferences. A recent systematic review highlighted that co-designed interventions for PEH improve mental health and housing stability, reduce hospital and emergency department admissions, and increase primary care utilisation, underscoring the promising nature of such approaches in providing continued access to healthcare [16]. Given the existing gaps in cancer care for PEH, the co-designed HNM is the first international study incorporating their voices to facilitate equal access to cancer prevention, including screening services, thereby addressing critical gaps in healthcare delivery for this group. Moreover, our results encompass findings from four different European healthcare systems and settings and identify overarching themes by contextualising the ten core components of navigation interventions delineated by DeGroff et al. [20]. This contextualisation facilitates the development of the HNM to be adaptable and scalable across Europe, with the aim of being effective in diverse healthcare settings.

Specifically, patient navigation programs are crucial in enhancing cancer prevention, particularly for PEH, as stated in a recent scoping review [4]. These programs focus on increasing adherence and participation in breast, cervical, and colorectal cancer screenings [23,24]. This is also highlighted in disadvantaged populations [15] and, more specifically, in PEH [5]. By embedding the HNM int places familiar to PEH, such as shelters or community centres, our suggested approach ensures timely access to preventive services. Coordinating medical and social services through innovative and multidisciplinary approaches such as HNM is a potential way to reduce the burden of cancer for this population [25].

A systematic review by Ali-Faisal et al. [26] supports the co-design findings, showing that both lay and professional patient navigators can be practical. However, while lay patient navigators are valuable, involving professionals such as social workers in navigation programs has a highly positive impact [27]. Their role in performing navigation tasks or providing hands-on support through training or supervision enhances outcomes. Therefore, training and education for those adopting the navigation role are crucial. However, an accredited certification for navigation still needs to be developed for professionals in this role or those supervising peer navigation programs.

Further research is needed to evaluate the long-term impact and effectiveness of the HNM in mitigating health inequalities and improving cancer outcomes for PEH across Europe. With the Europe Beating Cancer Plan, we have the opportunity to ensure an integrated care perspective is adopted from the start, maximising the opportunities of integrated working across health and social care to reduce health inequalities. As this study was conducted during the co-designing stage of the CANCERLESS project, the findings have been directly integrated into the project’s implementation and evaluation phase. These results are crucial for future steps, as they inform how the HNM can be adapted to meet the specific needs of both PEH and healthcare professionals, ensuring the model’s relevance and sustainability.

## Strengths and Limitations

The study involved a large interdisciplinary and international consortium, including academic institutions, public health and social care agencies, and NGOs supporting PEH, providing a broad range of perspectives on healthcare challenges and the design of the intervention. Focus groups were used as a key qualitative method to facilitate collaboration and gather detailed insights from a diverse group of participants, including PEH, healthcare and social care providers, and service managers. This approach allowed for rich, co-created input and the negotiation of meanings, which were crucial in shaping the HNM to reflect both the lived experiences of PEH and the professional realities of service providers. The involvement of individuals living in shelters, who provided feedback on the design, delivery, and protocols of the study, ensured their perspectives directly influenced the intervention. Follow-up sessions to discuss evaluation results helped foster a sense of ownership among participants, improving their engagement with the process [28]. However, the study primarily included people residing in shelters and frequent users of social services, which may have skewed the findings by underrepresenting rough sleepers or those with little to no contact with social services. This limitation in the representativeness of PEH participants highlights the need for broader inclusion of participants from more diverse groups of PEH to better inform the model’s design. While integrating patient navigation and empowerment offers a strong foundation for the HNM, the representation in our co-design groups may not fully capture the diversity of healthcare systems across Europe, potentially limiting the generalisability of the proposed model.

## Conclusions

The co-design of the HNM presented in this article reflects a participatory study conducted across Austria, Greece, Spain, and the UK. Following a theoretical approach, we provide a detailed description of the model through guided discussions and analysis. Our presentation not only encompasses key national findings but also identifies commonalities, enabling us to propose a model potentially adaptable to diverse health and care systems across Europe. Co-design processes at an international scale are inherently complex and require all stakeholders’ involvement. Ultimately, we advocate for the adoption of our integrated cancer care model for PEH to prevent cancer and combat health inequalities and reduce the cancer burden for this disadvantaged group.

## Data Accessibility Statement

The datasets generated and/or analysed during the current study are available in pseudonymised form from the corresponding author upon reasonable request.
